# Suppression of mutually incompatible proprioceptive and visual action effects in tool use

**DOI:** 10.1371/journal.pone.0242327

**Published:** 2020-11-18

**Authors:** Marvin Liesner, Wilfried Kunde

**Affiliations:** Department of Psychology, University of Würzburg, Würzburg, Germany; University of Bologna, ITALY

## Abstract

Movements of a tool typically diverge from the movements of the hand manipulating that tool, such as when operating a pivotal lever where tool and hand move in opposite directions. Previous studies suggest that humans are often unaware of the position or movements of their effective body part (mostly the hand) in such situations. It has been suggested that this might be due to a “haptic neglect” of bodily sensations to decrease the interference of representations of body and tool movements. However, in principle this interference could also be decreased by neglecting sensations regarding the tool and focusing instead on body movements. While in most tool use situations the tool-related action effects are task-relevant and thus suppression of body-related rather than tool-related sensations is more beneficial for successful goal achievement, we manipulated this task-relevance in a controlled experiment. The results showed that visual, tool-related effect representations can be suppressed just as proprioceptive, body-related ones in situations where effect representations interfere, given that task-relevance of body-related effects is increased relative to tool-related ones.

## Introduction

When we move our body, we typically do so for a reason. More precisely, we move to change what we perceive in a specific way. Sometimes such intended perceptual changes relate to the body itself. For example, when exercising or pantomiming, a person may just aim to move the body in a certain way, and observe her- or himself doing so. In most cases, though, humans aim to change the environment beyond their own body, such as when transporting an object from one place to another, or opening a door that was closed a moment before.

Body movements typically come with various perceptual changes for obvious reasons. All body-external changes are produced by some body movement, which inevitably goes along with some body-related perceptual changes as well. For example, when transporting an object from one place to another the actor not only perceives the movement of the object itself but he or she also sees and feels the movement of the corresponding effector bringing this object movement about. William James termed these inevitable perceptual changes “resident” effects of body movements [1]. Second, many of our body movements consistently produce changes in the environment, even when actors might not specifically aim to produce such body-external changes. For example, exercising in the sunlight naturally produces a corresponding movement of the shadow of the moving body, although the movement of the shadow was not the reason for moving. James called these body-external perceptual consequences “remote” movement effects [1]. It should be noted that other authors sometimes use different labels for these effects such as, for example, “body-related” vs. “body-external”, “proximal” vs. “distal” or “internal” vs. “external” with sometimes slightly different meanings and connotations. We chose here to stick with William James’ terminology because it reflects the crucial difference between the effects we studied here: namely, effects “residing” in or on the biological body and effects, which are “remote” and thus detached from the biological body.

Given the variety of perceptual changes that come with any given body movement, the question arises which of these changes should be mentally represented and attended to when it comes to generate and monitor such movements (representational weight) [2–4]. At first glance, there is no obvious reason to take into account and attend at all a certain resident effect when aiming at a certain remote effect, or conversely, to take into account and attend a certain remote effect when just aiming at a certain resident effect. Not attending, or “neglecting”, currently task-irrelevant movement effects, might be particularly important when remote and resident effects are mutually inconsistent to each other. For example, when you turn your computer mouse upside down and try to move the cursor from left to right, it is best to focus on this intended remote effect and to neglect that the hand has to move, and of course feels doing so, from right to left. Not “neglecting” the spatially inconsistent movement of the hand in such a situation can in fact create all kinds of performance costs [5–12].

There is evidence for “haptic neglect” [13] in case of such inconsistencies between resident and remote movement effects [14–17]. In one study [16] participants had to trace a dot performing circle movements on a screen with a cursor by moving their hand which was occluded from vision on a plane tablet in front of the screen. Crucially, the participants’ hand movements were not transformed into identical movements of the cursor. Instead, to produce circular cursor movements elliptic hand movements with either horizontal or vertical deviations from the “standard” circle were required. After every trial, participants had to indicate whether they believed they had drawn horizontal or vertical ellipses. The results showed that even with large deviations (with x-y ratios between 1:1.29 and 1:1.86 depending on conditions), participants could not reliably judge the orientation of the ellipses anymore. Moreover, haptic neglect seems to actually facilitate performance when there are spatial inconsistencies between proprioceptive and visual movement consequences.

Another instance where this occurs is mirror drawing, where participants have to copy a drawing but can only see a mirror image of their hand. Here a felt movement of the hand to the right produces a visual image of the hand moving to the left. Interestingly both, participants with permanently removed body perception (i.e. deafferented patients [18]) or transiently reduced body perception (because of rTMS over somatosensory cortex [19]), outperform neurotypical participants. Of course, it is unlikely that resident effects are ignored completely when performing a tool task given their high biological importance. For example, a person operating a tool should still notice when his or her hand is approaching a hot stove or bumping into another object. Indeed, it could be shown that despite focusing attention on the effective end of a tool, some attention still remains on the body part controlling the tool [20]. Furthermore, the previously mentioned performance costs when resident and remote effects are inconsistent [5–12] could not occur if people would always engage in “full” haptic neglect.

Nevertheless, there is accumulating evidence for haptic neglect when producing remote object movements that are spatially incompatible to movements of an operating body effector while the possibility of the opposite mechanism, an attenuation of conflicting remote object movements and conversely a focus of attention on the resident action effects has sparsely been considered so far. We are aware of two studies doing so. In one study [21] participants were asked to move their occluded hand to the left or right, which then foreseeably produced a movement of a cursor on a screen in either the same or the opposite direction. The response time increase for generating hand movements, which produced opposite rather than same movements of the cursor were reduced, though not abolished when participants were asked to ignore rather than attend to the cursor movement. This finding suggests some instruction-based control over the degree of processing remote movement effects in the course of generating a movement prior to actual movement execution though it also shows that even when asked to ignore visual action effects, these must still be attended to some degree.

Another study suggests that increased task-relevance of resident effects (and decreased importance of the remote effects) prompts attenuation of processing visual information after movement execution [9]. In this study, participants had to estimate the position of their visually occluded hand after performing hand movements that were transformed into either spatially compatible or spatially incompatible cursor movements on a screen. The usual finding in such paradigms is that spatial judgments of body effector and external object are biased towards each other suggesting an integration of the two to a certain degree (*spatial binding*, e.g. [22]). The results of this study showed an absence of this spatial binding effect when hand movements were transformed into spatially incompatible cursor movements, which accords with the idea that the representation of the visual, remote action component (the cursor) was given less weight in the incompatible conditions when participants were asked about their proprioceptive, resident effects.

To summarize, it has frequently been shown that incompatibility, for example in movement direction, of a controlled object and the effective body part controlling that object leads to performance deficits because of the conflicting associated effect codes [6–12]. The idea of neglecting or suppressing one of the two conflicting components to reduce these difficulties seems a plausible assumption. Previous research has mainly argued in favor of a haptic neglect of the resident effects in such situations and a coding of one’s actions mainly through remote codes. Such haptic neglect renders position estimates of body effectors less accurate and biased towards the position of a controlled remote object [13–16]. Haptic neglect appears functional in most tool-use tasks because it relates to a component of movement feedback that is of limited task relevance and thus it might be the “default” state when dealing with a spatially discrepant tool. Thus, it seems plausible that when operating a tool to produce a certain remote effect, which also produces an incompatible resident effect, one might focus one’s attention mainly on the remote effect to decrease the impact of conflicting sensory information and of divided attention. Recent research suggests though, that an increase of the relevance of resident action effects might lead to a reversal of this imbalance in perceptual precision [9]. This raises the question whether it is the nature of resident action effects per se or their reduced task-relevance that renders it subject to such neglect.

Both approaches suggest that when performing an action, which produces mutually incompatible resident and remote effects, one of these effects might be suppressed or attended to less and the other one might be enhanced or attended to more to reduce the conflict emerging from the anticipation and observation of these interfering action effects. Previous studies have suggested that resident effects are the ones that might be more prone to be suppressed in such situations [14–17]. Possible reasons for this are, for example, that such haptic neglect helps to maintain a feeling of control over the tool and flexibly adapt to new situations [16] or that the saliency of the visual, remote effects might make a coding and attending of actions through remote effects more likely than through resident effects [23]. Furthermore, it has been shown that visual feedback plays an important role when learning visuomotor transformations, thus stressing the importance of remote effects when they are spatially discrepant to resident effects [24]. These findings might suggest a general bias towards perceiving remote effects relatively unaltered and neglecting resident effects whenever they are interfering. However, one factor which has never been investigated in a controlled fashion to our knowledge, though its importance has been noticed [e.g., 16], is the factor of task relevance of the remote effects in situations commonly studied. When operating a remote tool (e.g., when putting a nail in a wall with a hammer), the effects on that tool are obviously more relevant for achieving the task than the resident effects on the body so they should obviously also be in the focus of attention of the acting individual. Distinguishing these factors might seem artificial since tools are almost always used to achieve a certain distal effect, which is thus task-relevant. However, other examples (e.g. the shadow example from the beginning) show that the relevance of resident and remote effects can indeed vary in other real-world contexts. Additionally this differentiation would give interesting insights into motor planning processes.

With this study, we aimed to show that both, remote action effects and resident effects can be equally neglected to overcome potential interference between them. Specifically, we manipulated the compatibility between resident and remote effects and their task-relevance independently (for details see *Procedure and task*). We expected that the precision of sensory information decreases with decreasing task-relevance of the respective effect component. This decrease of perceptual accuracy would indicate the “neglect” of the respectively less relevant effect component, i.e. a shift of attention away from this component. This would be an economic way of processing only the more relevant perceptual information to a larger extent given the observed division of attention between resident and remote effects in past research [20, 21, 25]. More importantly, we expected the decline of perceptual precision for the less relevant effect component to be more pronounced when resident and remote effects were incompatible because the contradiction of visual and proprioceptive effects should boost the necessity for this representational weighting mechanism. This would support the idea that neglect of one effect component, preferably the less relevant one, is involved in resolving interference between conflicting remote and resident effect components.

## Materials and methods

### Ethics statement

The project was approved by the ethics committee of the Institute for Psychology of the University of Würzburg under the reference number GZEK 2018–33. All procedures were in line with the Declaration of Helsinki.

### Participants

30 participants were tested and received 20 € for their participation. The number of participants was limited by laboratory capacities. Participants were recruited through an online platform used by the University of Würzburg and gave informed consent prior to the experiment. Due to the relatively long testing sessions (~ 120–135 minutes), four participants decided to cancel the testing before completion. Another two participants had to be excluded due to technical malfunctioning so that the final sample of participants consisted of 24 participants (all right-handed; 18 female, 6 male; *M*_*Age*_ = 25.67, *SD*_*Age*_ = 4.43, *min*_*Age*_ = 20, *max*_*Age*_ = 33). All participants had normal or corrected to normal sight and did not report any relevant medical conditions.

### Apparatus and stimuli

**Fig 1:**
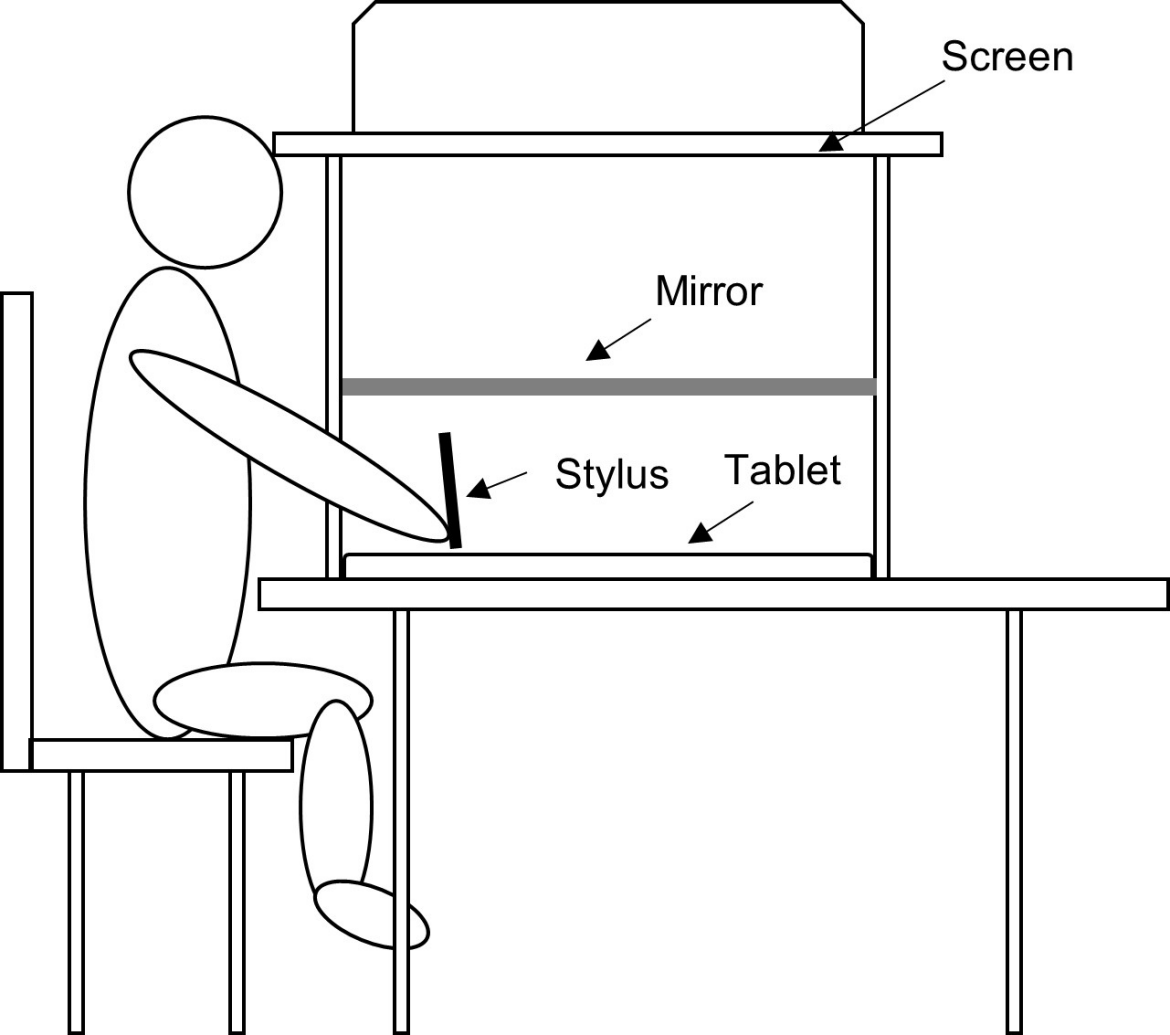
Experimental set-up as used in the study.

The experimental set-up is depicted in Fig 1. The apparatus consisted of a graphics tablet (Intuous 4 XL, Wacom, Kazo, Saitama, Japan) which was horizontally fixated on a table and an iron construction around the tablet holding a semi-silvered mirror approximately 26.9 cm above the tablet and a 24” LCD screen facing down on the mirror approximately 48.4 cm above the tablet. Furthermore, the computer keyboard was placed left of the construction. The experimental procedures were programmed using Eprime (version 2.0, https://www.pstnet.com/). Because the monitor was slightly larger than the graphics tablet, the projection area of the monitor was adjusted so that it resembled the measures of the tablet to ensure that the screen image was projected onto the mirror which gave the impression for participants that a screen of just the same size and dimensions as the tablet was mounted above the tablet. One pixel was approximately 0.25 x 0.25 mm^2^ in size. Participants were seated in front of the construction and were instructed to lean their forehead against the upper part of the construction facing down at the mirror. Additional black cardboard prevented vision of the computer screen. Participants placed their right hand between the tablet and the mirror and were able to operate the tablet with a digitizing stylus. They were instructed to place their left hand left of the construction so that they were able to operate the keyboard with it. Because vision of the keyboard was hindered due to the set-up, all relevant keys for the experiment (arrow keys, space bar, return key) had little soft markers attached to them so that they could be easily identified without vision. To ensure that participants could not see their hand on the tablet through the mirror, all testing was carried out in a darkened room.

### Procedure and task

**Fig 2:**
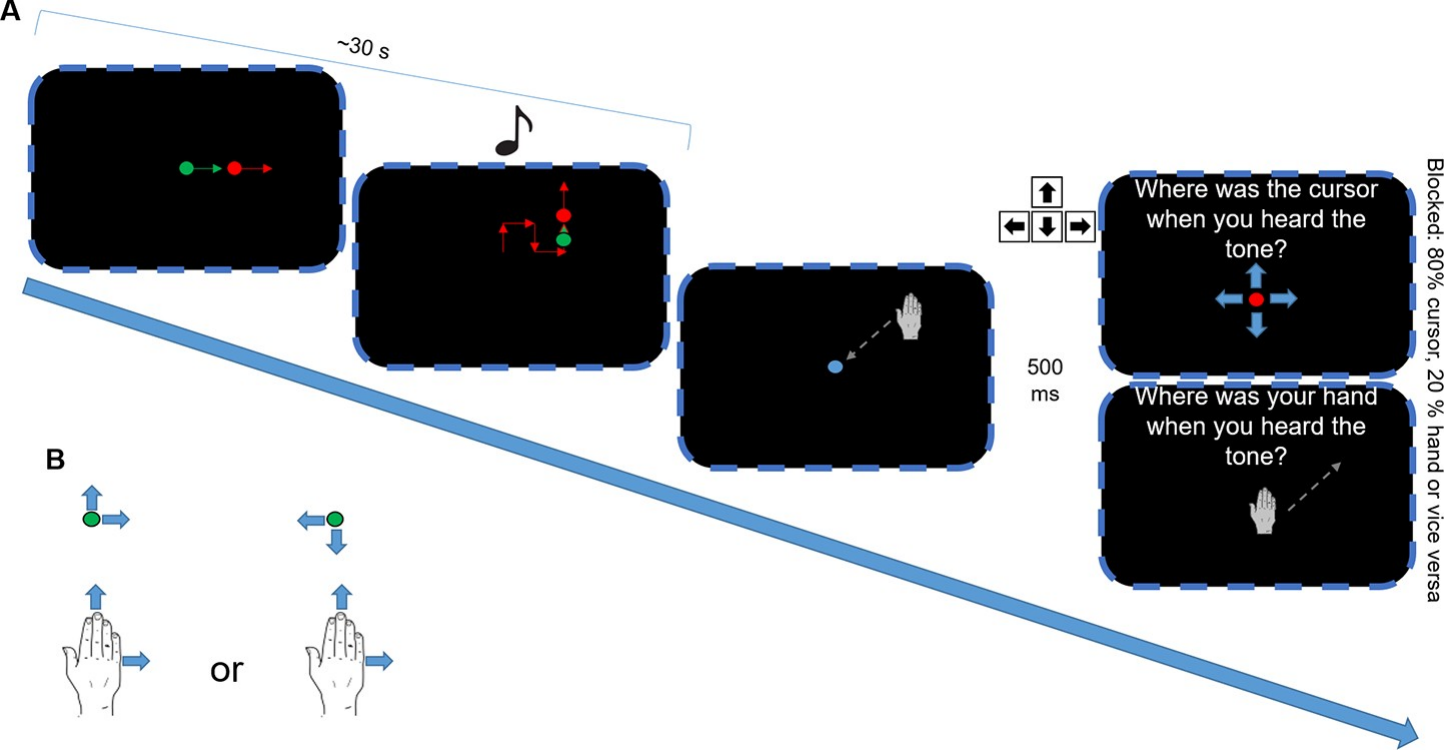
(A) Trial structure of the experiment. Please note that the depicted hand was not visible to the participants since it was placed beneath the projection area as shown in Fig 1. (B) Hand-cursor movement transformations in the compatible (left) and incompatible (right) conditions.

Each trial included the following steps for the participants (also depicted in Fig 2). First, participants had to move a red cursor (a dot approximately 0.5 mm in diameter) controlled with the stylus to the center of the screen, which was marked by another green dot equal in size. After a short random delay (1000 ms or 2000 ms, counterbalanced across all conditions), a beep tone (50 ms, 2000 Hz) was played as a start signal and the green dot started moving in one out of four directions (up, right, down, left) chosen randomly in a straight line of a length chosen randomly within a range of 12.5–37.5 mm with a constant speed of 0.5 mm per frame. After this distance was covered by the dot, the experiment generated another random segment in one out of the four directions and within the same range. This procedure was repeated until a number of segments between 5 and 15 was reached. Again, also number of segments was determined on a random basis within this range in every trial. This procedure resulted in the green dot moving on the screen continuously taking several 90° or 180° turns for about 30 s per trial. Throughout the whole dot movement phase, the participants’ task was to follow the green dot as accurately as possible with the cursor by moving the stylus on the tablet. If the distance (measured as Euclidean distance) between the green dot and the cursor exceeded 10 mm, an error message was shown and the trial was repeated. During the last segment of every trial, another short tone (50 ms, 500 Hz) indicated that the trial was about to end after this segment.

After the last segment had ended, both the green dot and the red cursor disappeared and participants had to move the stylus back to the center of the tablet as marked by a blue dot. After the center had been approached and an additional delay of 500 ms, the second, judgment phase of the trial started in which participants were asked to estimate either the position of the red cursor or the position of the stylus at the moment when the second tone occurred which had signaled the end of the trial. In cursor judgment trials, the red cursor appeared on the screen again and it had to be moved to the estimated position by the participants by using the arrow keys of the keyboard with their left hand and confirming their judgment with the return key. In stylus judgment trials, no cursor was shown but the participants were simply asked to move the stylus back to the estimated position and confirm their judgment by pressing a key on the stylus. After one of the two judgments had been made, the next trial started.

The experiment was split into four blocks, which differed in the way the participants’ movements of the stylus were transformed into movements of the cursor and in the ratio of stylus judgment and cursor judgment trials (see Table 1). In *compatible* blocks, the cursor always moved in the same direction as the participant moved their hand, thus a movement of the hand to the left caused a cursor movement to the left, an upwards hand movement caused an upwards cursor movement etc. In *incompatible* blocks, the direction of cursor movements was always inverted both in the x and in the y direction compared to the hand movements. Thus, a hand movement to the left caused a cursor movement to the right, an upwards hand movement caused a downwards cursor movement etc. Furthermore, the blocks differed in terms of the frequency of cursor and stylus judgments. In *mainly proprioceptive* blocks, 80% of the judgments that had to be made were stylus judgments and only 20% were cursor judgments whereas in *mainly visual* blocks this proportion was the other way round. Please note that the order of judgments in a given block was still randomized so that only the ratio, but not the sequence of certain judgments could be predicted. Additionally, there were no explicit instructions for the participants regarding the judgment ratios. The manipulations of compatibility and judgment ratio were combined leading to the blocks *compatible x mainly proprioceptive*, *compatible x mainly visual*, *incompatible x mainly proprioceptive* and *incompatible x mainly visual* (see Table 1). Participants always either completed both compatible or both incompatible blocks first before experiencing the second movement transformation and furthermore underwent short practice blocks (8 trials) before both the compatible and incompatible half of the experiment. In these practice blocks, the percentage of cursor judgments and hand judgments was 50% each. Whether participants first performed the compatible or incompatible blocks and order of judgment ratio blocks within the compatible and incompatible blocks was counterbalanced between participants.

**Table 1:** Blocks used in the experiment combining the manipulations of hand-cursor transformation and judgment proportion.

Hand-cursor transformation	Judgment proportion
Compatible	80% cursor, 20% hand
Compatible	20% cursor, 80% hand
Incompatible	80% cursor, 20% hand
Incompatible	20% cursor, 80% hand

Participants always underwent either both compatible or both incompatible trials before experiencing the second transformations. Order of transformations and order of blocks within transformations were counterbalanced.

Furthermore, we varied the time point of the occurrence of the tone signalizing the end of the trial. The tone could occur either when the cursor was still 2.25–2.5 mm away from the end of the last segment (we will refer to that from now on as *very late*), when it was 4.75–5 mm away from the end of the last segment (*late*), when it was 7.25–7.5 mm away from the end of the last segment (*early*) or when it was 9.75–10 mm away from the end of the last segment (*very early*). We varied this time point to prevent participants from developing strategies for their judgments, which they could possibly do if the continuation of the movement after the tone occurred had always been of the same length. In combination with the variable start delay of either 1000 ms or 2000 ms which was counterbalanced over all other conditions, participants thus performed 40 trials per block leading to a total number of 176 trials including practice blocks and plus potential additional trials to replace error trials. Within blocks, trials were randomized.

### Data preprocessing

We excluded all practice trials and trials in which participants deviated more than 10 mm at any time, measured as Euclidian distance, from the green dot they had to track with the cursor. For every trial, we calculated the absolute error of the judgments as the Euclidian distance between the actual position of the to-be-judged entity (i.e. the cursor or the stylus) at the moment of sound occurrence and its estimated position by the participant.

## Results

All raw data and analysis scripts are available on the Open Science Framework, osf.io/kmbe3/.

Median absolute errors were entered into a 2 x 2 x 2 x 4 repeated measures analysis of variance (RM ANOVA) with the factors judgment modality (proprioceptive vs. visual), compatibility (compatible vs. incompatible), judgment frequency (high vs. low) and time point of tone (very late vs. late vs. early vs. very early). Furthermore, exploratory Bayes Factors were estimated for all reported main effects and interactions. As expected, absolute errors were larger for the incompatible than for the compatible movement transformation (*M*_*incompatible*_ = 34.81 mm, *SD*_*incompatible*_ = 23.25 mm; *M*_*compatible*_ = 20.08 mm, *SD*_*compatible*_ = 8.92 mm; *F*(1, 23) = 18.80, *p* = .001, *η*_*p*_^*2*^ = .37; *BF*_*10*_ = 1.27 x 10^13^) and for the less frequently judged modality than for the more frequently judged one (*M*_*low*_ = 31.69 mm, *SD*_*low*_ = 17.82 mm; *M*_*high*_ = 23.20 mm, *SD*_*high*_ = 11.85 mm; *F*(1, 23) = 28.81, *p* < .001, *η*_*p*_^*2*^ = .56; *BF*_*10*_ = 2.93 x 10^3^).

**Fig 3:**
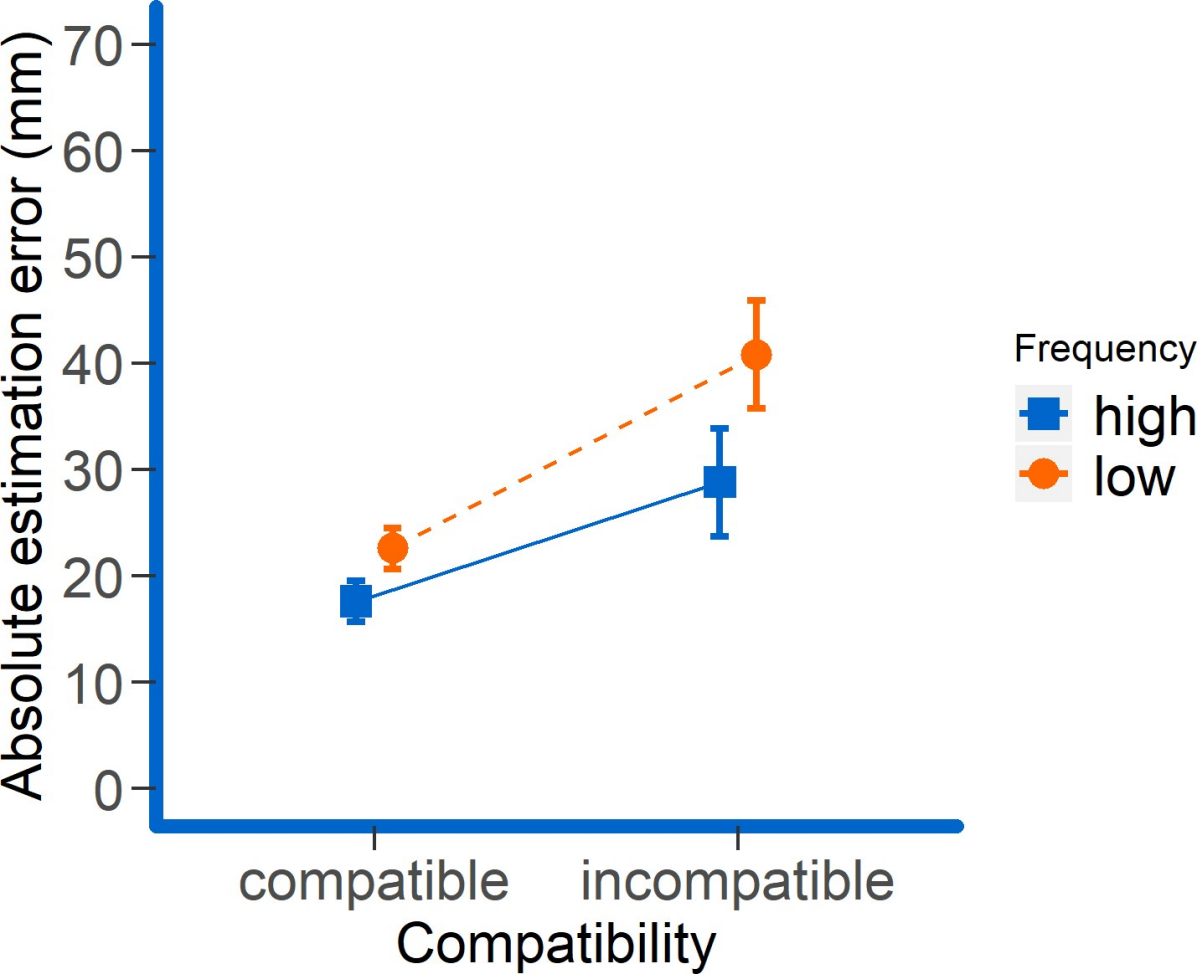
Mean absolute errors as a function of compatibility and frequency of the judgment. Error bars represent 95% paired-difference confidence intervals [26].

**Fig 4:**
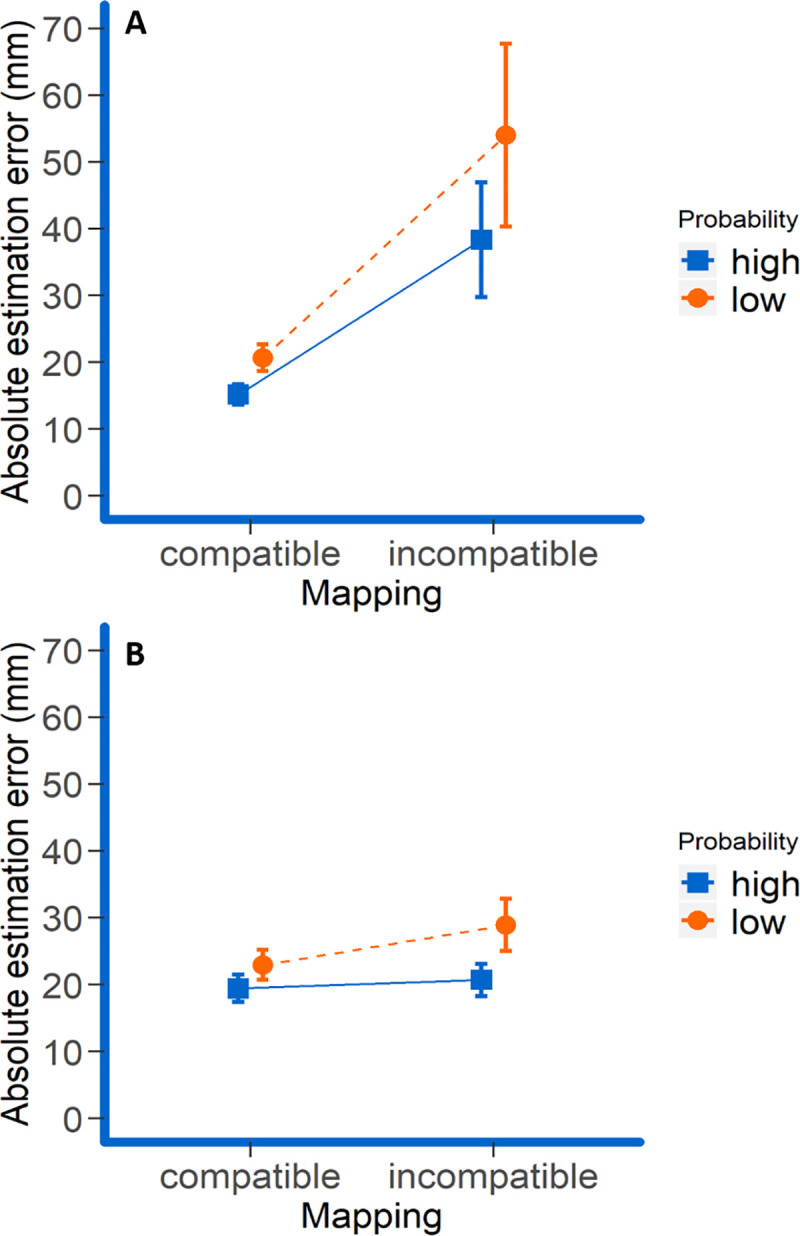
Mean absolute errors as a function of compatibility and frequency of the judgment split up for stylus judgments (A) and cursor judgments (B). Error bars represent 95% paired-difference confidence intervals [26].

These effects were further modulated by the significant two-way interactions compatibility x judgment modality (*F*(1, 23) = 8.93, *p* = .007, *η*_*p*_^*2*^ = .28; *BF*_*10*_ = 4.38 x 10^11^) and compatibility x frequency (*F*(1, 23) = 4.94, *p* = .036, *η*_*p*_^*2*^ = .18; *BF*_*10*_ = 0.91) although for the latter one the Bayes factor suggested inconclusive evidence. Follow-up t-tests for the compatibility x judgment modality interaction indicated that the difference between absolute errors in the compatible and incompatible condition was only significant for proprioceptive but not for visual judgments (*M*_*compatible x visual*_ = 22.16 mm, *SD*_*compatible x visual*_ = 12.55 mm; *M*_*incompatible x visual*_ = 24.11 mm, *SD*_*incompatible x visual*_ = 10.82 mm; *|t|*_*visual*_ < 1; *M*_*compatible x proprioceptive*_ = 17.92 mm, *SD*_*compatible x proprioceptive*_ = 6.70 mm; *M*_*incompatible x proprioceptive*_ = 45.52 mm, *SD*_*incompatible x proprioceptive*_ = 42.55 mm; *t*_*proprioceptive*_(23) = 3.48, *p* = .002, one-tailed, *d*_*z*_ = .71, adjusted for multiple testing). More importantly, the compatibility x frequency interaction indicated that the observed frequency effect was larger in the incompatible than in the compatible condition (*M*_*high x compatible*_ = 17.59 mm, *SD*_*high x compatible*_ = 8.10 mm; *M*_*low x compatible*_ = 22.57 mm, *SD*_*low x compatible*_ = 10.42 mm; *M*_*high x incompatible*_ = 28.81 mm, *SD*_*high x incompatible*_ = 18.47 mm; *M*_*low x incompatible*_ = 40.82 mm, *SD*_*low x incompatible*_ = 29.07 mm; see Fig 3) though the frequency effects were significant in both compatibility conditions (both *t*’s > 4.05, both *p*’s < .001, one-tailed, adjusted for multiple testing). This effect was not further modulated by the three-way interaction compatibility x frequency x judgment modality (*F*(1, 23) = 1.20, *p* = .29, *η*_*p*_^*2*^ = .050; *BF*_*01*_ = 3.88) suggesting that this pattern was independent of the to-be-judged entity. Thus, the results suggest that in the case of incompatible hand and cursor movements, the judgment error is specifically increased for the less frequently judged modality, regardless of whether this is the visual or proprioceptive modality (Fig 4). Neither the main effects of judgment modality and time point of tone nor any other interactions reached significance (all *p*s > .059).

Due to the relatively low number of trials for estimating the median values for some of the cells (especially in the low frequency condition), we also conducted an analysis based on linear mixed models (LMMs) which does not require the aggregation over trials like the RM ANOVA. The results largely confirmed those of the ANOVA with the only difference that the main effect of judgment modality reached significance in the LMM analysis (*t* = 10.46, *p* < .001). Most importantly though, the fit of the main effects model was improved significantly by adding the two-way interaction of compatibility x frequency (*χ*^*2*^(1) = 5.91, *p* = .015) and the fit of the two-way interaction model was not improved further by adding the three-way interaction of compatibility x frequency x modality (*χ*^*2*^(2) = 3.06, *p* = .22). This shows that the observed results were not due to properties of particular analysis methods.

**Fig 5:**
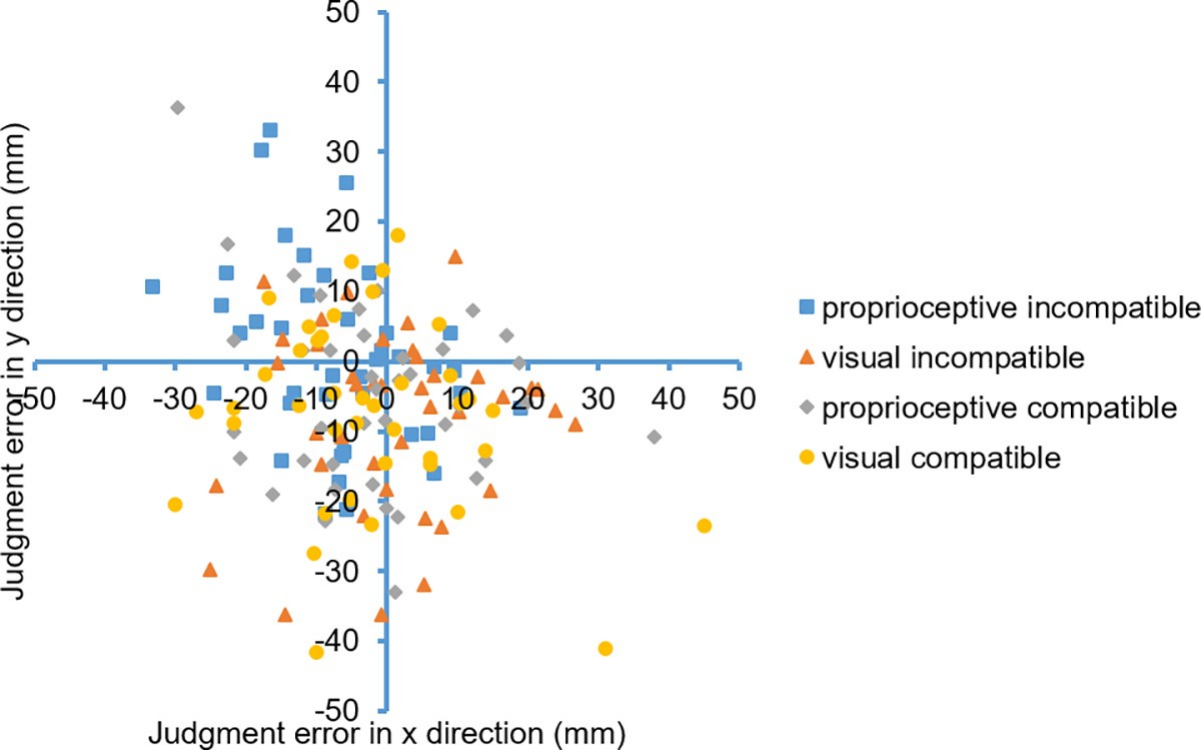
Example data from one participant (participant 9) showing the deviation from the to-be-judged position in x and y direction. Target and judgment positions for all trials were remapped to the upper left quadrant of the screen so that data points in the right and lower half of the figure represent judgment errors towards the center and data points in the left and upper half of the figure represent judgment errors towards the sides.

As an example, Fig 5 shows the judgment data of a typical participant.

### Exploratory analyses

To assess whether our paradigm is able to trigger a default haptic neglect without making one of the two action components more or less task-relevant, we calculated an additional 2 x 2 RM ANOVA on median absolute errors in the practice trials with the factors judgment modality (proprioceptive vs. visual) and compatibility (compatible vs. incompatible). We did not include the factor time point here because it did not reveal any significant effects in the previous analysis. We found significant main effects of both factors (modality: *F*(1, 23) = 7.10, *p* = .014, *η*_*p*_^*2*^ = .27, *BF*_*10*_ = 23.48; compatibilty: *F*(1, 23) = 17.98, *p* < .001, *η*_*p*_^*2*^ = .44, *BF*_*10*_ = 397.06), but more importantly also a significant interaction between the two (*F*(1, 23) = 13.05, *p* = .001, *η*_*p*_^*2*^ = .36, *BF*_*10*_ = 1.59 x 10^3^). Follow-up t-tests revealed that this was due to significantly less precise proprioceptive judgments in the incompatible compared to the compatible condition (*M*_*compatible x proprioceptive*_ = 22.46 mm, *SD*_*compatible x proprioceptive*_ = 8.74 mm; *M*_*incompatible x proprioceptive*_ = 57.88 mm, *SD*_*incompatible x proprioceptive*_ = 40.58 mm; *t*(23) = 4.86, p < .001, *d*_*z*_ = .99), but that there was no such effect in visual judgments (*M*_*compatible x visual*_ = 23.71 mm, *SD*_*compatible x visual*_ = 12.59 mm; *M*_*incompatible x visual*_ = 26.53 mm, *SD*_*incompatible x visual*_ = 25.38 mm; |*t*| < 1). Furthermore, proprioceptive judgments were only less precise than visual ones in the incompatible (*t*(23) = 3.23, p = .004, *d*_*z*_ = .67), but not in the compatible condition (|*t*| < 1).

## General discussion

In this experiment, we investigated how the task-relevance of resident and remote action effects (as manipulated by probability of probing) and level of interference (as manipulated by mutual (in)compatibility between them) impacts the precision with which these action effects are perceived. We found that perceptual precision was generally lower for the effects that were less likely probed, and when resident and remote effects were incompatible to each other. This suggests that people are generally sensitive to task demands when it comes to shift attention to either resident effects of their actions or effects located in a remote, controlled object. Most notably, the drop of judgment accuracy was particularly pronounced for less relevant, incompatible action effect components. This suggests that “neglect” of task-irrelevant effect components might be involved to overcome interference that would otherwise occur by attending to these effect components. Importantly, this occurred both when the cursor position was less likely to be probed indicating the neglect of interfering visual information when proprioceptive judgments were more likely, and when the stylus judgment was less likely to be probed indicating the neglect of interfering proprioceptive information (i.e., *haptic neglect*) when visual judgments were more likely (Figs 3 and 4). The corresponding interaction was significant in the ANOVA and the linear mixed model analyses, while the Bayes analysis yielded inconclusive evidence for this interaction. So a higher-powered study of this effect is certainly desirable.

Previous studies on tool use have argued that visual attention is focused on the effective end of the tool rather than the person’s body movements [16, 17, 20]. The present study however showed that this mechanism is shaped by the relevance of the resident sensations while performing a tool task. Heuer & Rapp [13] have suggested that interference between action effect components is reduced by neglecting proprioceptive information. Here we showed that not only proprioceptive but also remote information can be suppressed to reduce conflict. It is thus not the particular nature of proprioceptive action effects that makes them subject of such suppression. Instead, this happens because of their irrelevance in tasks where remote effects, such as movements of a tool, are the goal of the action [14–16]. In line with previous work [13, 16], our exploratory analyses also showed that without the upweighting of proprioceptive information through increased relevance of resident signals these are more likely to be ignored or suppressed than remote visual information about the tool when the information from these sources is contradictory. One might wonder why the absolute errors were larger with a 50% chance of being probed in the practice blocks than in the 20% chance condition in the experimental blocks. This is most likely due to practice effects with the judgment task. However, this inflation in the practice trials should not affect the critical comparisons. In line with these exploratory analyses, we also found no general compatibility effect in precision judgments for the visual condition in absence of further manipulations of task-relevance. Both observations suggest that there is no “vision neglect” under normal tool use conditions, which makes sense given the inherent importance of the tool and its effects for achieving one’s goal when using a tool [13, 16]. As laid out before, to invert this tendency proprioceptive information needs to become more task relevant.

Our findings thus suggest that despite a “default” tendency to neglect incompatible proprioceptive instead of visual information the mechanisms related to the processing of resident and remote effects still seem to be more similar than previous studies on tool use suggested. This is well in line with theoretical notions that humans should be very flexible in their way of representing actions either through anticipation and feedback from resident or remote action effects [3, 4]. There might be a more general mechanism underlying these observations, related to the processing of spatially incompatible sensory information of different modalities in general, independent of the stage of movement, or possibly of movements at all. We will come back to this possibility at a later point. To what extent participants are actually aware of these seemingly strategical suppression mechanisms is certainly something to be determined in future research.

As outlined above, the neglect of the less relevant action effects in a given block and the modulation of this through (in)compatibility of resident and remote effects was independent of the judgment modality, thus whether visual or proprioceptive estimates had to be made. Additionally though, we also found no significant differences between absolute estimation errors of the two judgment modalities in the experimental blocks which is surprising given the common finding that people are usually much better in judging visual compared to proprioceptive positions [22, 27–30], though we did find a significant effect of this factor in the exploratory single-trial analyses. However, this might be due to the significant crossed judgment modality x compatibility interaction which indicated not only a compatibility effect for the proprioceptive judgments, but also that these were both less precise than visual judgments in the incompatible condition, while being more precise than visual judgments in the compatible condition. Conceivably, the surprisingly precise proprioceptive judgments in the compatible condition resulted from moving the stylus back to where it had been during the occurrence of the tone. Thus, participants could have tried to move back from the center position to the estimated position in more or less the same trajectory in which they moved from the end position of the tracking task to the center position. We tried to circumvent this by introducing a delay before indicating the judgment modality and by varying the time point of the tone; however, it could still be that this experimental characteristic might have led to an advantage for the proprioceptive judgments, which was not available in the visual judgments. Another possible factor might have been that despite the disappearance of the cursor, the neutral center position of the cursor after every trial and the delay before judgments, the location of the visual information might have been more accurately remembered than the location of proprioceptive information [31]. Consequently, the stylus judgments might have been supported by additional visual information to a greater extent than the cursor judgments were supported by additional proprioceptive information.

Furthermore, in compatible trials cursor and hand were always right on top of each other throughout the tracking phase while this was not the case in incompatible trials. Thus, besides the (in)compatibility of the movement directions of hand and cursor, there was also an additional spatial discrepancy in the incompatible condition between them which might have additionally contributed to the distribution of attention between hand and cursor. As a result, the possible additional support of the proprioceptive judgments through visual information in the compatible conditions might have led to an underestimation of both the overall level and the compatibility effect of proprioceptive estimation errors. Nevertheless, such biases would have not affected the previously discussed main findings. On the contrary, that incompatibility affected uncertainty about proprioceptive sensations stronger than uncertainty about visual sensations, renders the suppression of conflicting visual signals of low task relevance even more remarkable.

An important point that should still receive some attention here is our manipulation of task-relevance through the frequency of probing a specific modality in the judgment task. While the relevance of the sensory modalities for the judgment task is obviously manipulated by this, the relevance of especially visual information during the tracking task might be more difficult to manipulate. The processing of the visual information from the cursor is essential in the tracking task since the task cannot be fulfilled based on proprioceptive information alone. Therefore, one might expect that participants should perform the two tasks as if they were independent of each other and there should be no carryover between the two tasks. However, this would render the judgment task impossible to do above chance performance, because the sensory information (visual or proprioceptive) necessary for the position judgment was only available during the tracking task and thus had to be attended and encoded during this task already for the subsequent judgment. Thus, if participants were to adapt to the different movement transformation mappings and the proportion of cursor or hand judgments, they necessarily had to process this sensory input differently already during the tracking task, which then showed up in the subsequent judgement task.

While it might seem contradictory, that especially visual information could already be suppressed during the tracking task, a way to solve this is to think of neglect of sensory information less in absolute or discrete ways, but rather as a continuous distribution of attentional resources between different sensory modalities [3, 4, 32–34]. Participants certainly could not completely neglect visual information in the less relevant condition because it was still necessary for the tracking task, nevertheless they might have already shifted away some attention from the visual information compared to the high relevance condition but leaving still enough to perform the tracking task. Thus, the manipulation of task relevance might still also have an effect on the degree to which visual information is already neglected in the tracking task, though the impact of the manipulation might have been smaller here than for the proprioceptive information. This might also explain the smaller visual effects in general and the tendency towards neglecting proprioceptive rather than visual information during practice trials and when leaving out the relevance manipulation. Possibly, this imbalance could be reduced if one chose a stronger manipulation of relevance of resident or remote effects already in the tracking task, like for example by making the tracking task dependent on haptic instead of visual guidance. However, this would have essentially undermined the purpose of this study to investigate perception of resident and remote effects under different movement transformations in tool use since tool use is characterized by manipulating an external object, in this case, a cursor. Thus, making the goal of the tracking task directly related to the resident effect itself would lead to a conceptually different situation. There might still be other ways to manipulate the relevance of resident and remote effects and at the same time maintain the nature of the tool use task, which might be worth to be tested in future research, however we believe to have provided one possible way to do so.

Discrepant sensory input of different modalities might be processed to different degrees without active movements. Therefore, an experiment similar to ours where locations of mutually (in)compatible visual and tactile stimulations have to be judged with varying probabilities might reveal similar results, without actively producing that input. Still, tool-use is a real world example where such a situation is inevitably created, while it needs to be studied further whether the active production and passive encounter of such discrepancy matters. Ideo-motor theory [1–3, 35] suggests that it would matter, as mutually incompatible sensory effects of motor patterns create a problem for producing these motor patterns in the first place, which adds a reason to suppress one of the two components already during action generation. An additional point that we shortly want to touch is a possible relationship between the two tasks and specifically whether differential performance on the tracking task might have influenced performance on the judgment task. While we did not have tracking data to control for this possibility, such possible influences could, if at all, only affect the main effect of compatibility in the judgment task. Since our main results however rest on more complex interactions with this factor, additional mechanisms, presumably the ones we suggested, have to be at play for these. Actually, since we suggest that these mechanisms are already present during the tracking task, they might also be the reason for possible performance differences in the tracking task

Furthermore, also the manipulation of time point of the tone that indicated the to-be-judged position of either hand or cursor should still receive some attention here. While the main reason for varying this time point was to prevent predictability of the to-be-judged position based on the movement endpoint, in principle this manipulation could have also had an influence on the absolute estimation error so that it could have made judgments for example more inaccurate, the earlier before the end of the movement it occurred and thus the more time had passed between the tone and the judgment. However, we did not find such an effect, either because the spatial judgments were insensitive to this manipulation or because the time points were chosen too close together.

## Conclusions

Controlling an external object through one’s own body movements can lead to discrepancies (e.g. spatial) between the body effector and the controlled object. Previous research has suggested that the conflict emerging from this can be overcome or reduced by focusing one’s attention on the effects in the object and attending away from one’s body. Implementing a paradigm, which manipulated the relevance of the object on the one hand and of the body on the other hand during the task showed that this attentional focus can also be reversed so that the body instead of the object is attended.
